# Immunomodulatory and Antioxidant Effects of Spray-Dried Encapsulated Kale Sprouts after *In Vitro* Gastrointestinal Digestion

**DOI:** 10.3390/foods12112149

**Published:** 2023-05-26

**Authors:** Erika Ortega-Hernández, Ana Victoria Camero-Maldonado, Laura Acevedo-Pacheco, Daniel A. Jacobo-Velázquez, Marilena Antunes-Ricardo

**Affiliations:** 1 Tecnologico de Monterrey, Escuela de Ingeniería y Ciencias, Centro de Biotecnología-FEMSA, Av. Eugenio Garza Sada 2501 Sur, Monterrey 64849, Mexico; erika_ortega@tec.mx (E.O.-H.); laura.acevedo@tec.mx (L.A.-P.); 2 Tecnologico de Monterrey, Institute for Obesity Research, Ave. Eugenio Garza Sada 2501 Sur, Monterrey 64849, Mexico; 3 Tecnologico de Monterrey, Escuela de Medicina y Ciencias de la Salud, Av. Ignacio Morones Prieto 3000, Monterrey 64710, Mexico; anav.camero@tec.mx; 4 Tecnologico de Monterrey, Escuela de Ingeniería y Ciencias, Ave. General Ramón Corona 2514, Zapopan 45201, Mexico; 5 Tecnologico de Monterrey, Institute for Obesity Research, Ave. General Ramón Corona 2514, Zapopan 45201, Mexico

**Keywords:** kale, germination, encapsulation, phytochemicals, cellular antioxidant activity, anti-inflammatory activity, immunomodulatory activity

## Abstract

The health-related compounds present in kale are vulnerable to the digestive process or storage conditions. Encapsulation has become an alternative for their protection and takes advantage of their biological activity. In this study, 7-day-old Red Russian kale sprouts grown in the presence of selenium (Se) and sulfur (S) were spray-dried with maltodextrin to assess their capacity to protect kale sprout phytochemicals from degradation during the digestion process. Analyses were conducted on the encapsulation efficiency, particle morphology, and storage stability. Mouse macrophages (Raw 264.7) and human intestinal cells (Caco-2) were used to assess the effect of the intestinal-digested fraction of the encapsulated kale sprout extracts on the cellular antioxidant capacity, the production of nitric oxide (NOx), and the concentrations of different cytokines as indicators of the immunological response. The highest encapsulation efficiency was observed in capsules with a 50:50 proportion of the hydroalcoholic extract of kale and maltodextrin. Gastrointestinal digestion affected compounds’ content in encapsulated and non-encapsulated kale sprouts. Spray-dried encapsulation reduced the phytochemicals’ degradation during storage, and the kale sprouts germinated with S and Se showed less degradation of lutein (35.6%, 28.2%), glucosinolates (15.4%, 18.9%), and phenolic compounds (20.3%, 25.7%), compared to non-encapsulated ones, respectively. S-encapsulates exerted the highest cellular antioxidant activity (94.2%) and immunomodulatory activity by stimulating IL-10 production (88.9%) and COX-2 (84.1%) and NOx (92.2%) inhibition. Thus, encapsulation is an effective method to improve kale sprout phytochemicals’ stability and bioactivity during storage and metabolism.

## 1. Introduction

Inflammation is a biological response to a biological, chemical, or physical factor present in the body that results in the breakdown of tissue homeostasis [1]. The pathophysiology of inflammation starts with amplifying of cellular oxidative stress mediated by high levels of reactive oxygen and nitrogen species. Subsequently, numerous immune cells are recruited, mainly macrophages, which promote the production of local proinflammatory mediators. This process is characterized by elevated levels of proinflammatory cytokines such as tumor necrosis factor-alpha (TNF-α), interleukin (IL)-1β, and IL-6, as well as decreased levels of anti-inflammatory cytokinin interleukin 10 (IL-10) [2].

When acute inflammation is not resolved correctly, a chronic inflammatory state is triggered with systemic repercussions. Thus, chronic inflammation has emerged as one of the key physiological mechanisms linking obesity with insulin resistance and diabetes, as well as being closely associated with the development of other serious pathologies, such as nonalcoholic steatohepatitis and cardiovascular diseases [3].

Currently, the treatment of inflammation is symptomatic, through the use of non-steroid anti-inflammatory medications (NSAIDs). However, numerous adverse side effects are associated with NSAIDs, including gastrointestinal issues, water retention, renal failure, bronchospasm, and hypersensitivity reactions [4]. Importantly, recent research indicates that lifestyle habits as well as the incorporation of some types of foods into the diet may protect against chronic inflammatory-related diseases. This has driven the interest in the search for functional ingredients that can prevent or mitigate the progression of inflammatory-related non-communicable diseases as an effective alternative to improve the health and quality of life of the population.

It is known that fruits and vegetables are good sources of vitamins, minerals, dietary fiber, and secondary metabolites that benefit our health, such as carotenoids, glucosinolates, and phenolic compounds. Their bioactive potential is due to phytochemicals’ capacity to modulate metabolic processes in the organism by neutralizing free radicals, activating enzymes, inhibiting cell receptors, and modulating gene expression [5].

Members of the Brassica genus (e.g., broccoli, cabbage, kale, cauliflower, and Brussels sprouts) are among the most health-promoting, widely cultivated, and widely consumed vegetables worldwide [6]. In particular, kale (*Brassica oleracea* L. var. acephala) is an economically significant crop. Young kale sprouts are an exceptionally abundant source of phenolic compounds, carotenoids, and glucosinolates, with concentrations several times higher than in kale leaves [7,8,9,10,11,12]. Likewise, different abiotic stress strategies have been used to increase the content of these phytochemicals, including salt stress [13,14,15]. Cruciferous plants can store sulfur and selenium in levels exceeding 100 μg g^−1^ of dry matter [16]. As the insufficiency of selenium in the diet of human beings is a prevalent issue worldwide, the propensity of some plants to accumulate selenium makes them a significant source of this nutrient for consumers [17].

However, environmental, and physiological factors such as oxygen, temperature, enzymatic hydrolysis, and pH changes directly affect the stability of phytochemicals and their bioavailability [18]. Therefore, it is necessary to protect these components by developing delivery systems to allow these molecules to remain stable during storage and gastrointestinal digestion, to favor their bioactivity.

Encapsulation is a technique that seeks to stabilize specific compounds for their application as nutraceuticals or in food-grade matrices, and to control their release in the gastrointestinal tract, thereby increasing their bioavailability [19]. Among the available encapsulation techniques, spray-drying stands out as a scalable and low-cost method that can form a stable, free-flowing powder [20]. Commonly, these phytochemicals are entrapped in a matrix of polysaccharides or proteins formed when atomized liquid meets heated air. In comparison to traditional drying procedures, the drying time is typically shorter (1–20 s) [20,21]. The effects of the spray-drying conditions and wall materials on the stability and encapsulation efficiency of glucosinolates and phenolic compounds in Brassicaceae vegetables has been previously reported [22,23,24,25]. Radünz et al. [22] reported the influence of encapsulation on broccoli extracts during digestive processes. The capsule mainly protected 5-caffeoylquinic acid, 4-hydroxybenzoic acid glucoside, *p*-coumaroylquinic acid, 4-methoxy-gluco-brassicin, 1-*O*-sinapoyl-β-D-glucose, and coumaric acid; at the same time, inhibitory activity for the nitric oxide radical showed an increase (74.8%) in the intestine fractions. Later, Radünz et al. [22] found that the encapsulation of broccoli extract showed greater protection of the glucosinolates and phenolic compounds (86.9%), without affecting the active sites against glial tumor cells. However, the effect of encapsulation on the phytochemicals of kale during gastrointestinal digestion and their bioactive properties has not been studied.

Therefore, in the present study, the human digestion of kale sprouts was simulated using an *in vitro* digestion model. This system was sampled at the mouth, stomach, and intestinal stages. The bioaccessibility of kale sprouts in each digest was determined, and the cellular permeability of the identified carotenoids, glucosinolates, and phenolic compounds was evaluated in Caco-2 cell monolayers differentiated into intestinal epithelial cells.

This study aimed to assess the antioxidant and immunomodulatory activity of encapsulated and non-encapsulated kale sprouts treated with S and Se after gastrointestinal digestion.

## 2. Materials and Methods

### 2.1. Chemicals

Ethanol (HPLC grade), sodium selenite (Se; Na_2_SeO_3_), potassium sulfate (S; K_2_SO_4_), sodium chloride (NaCl), potassium chloride (KCl), sodium bicarbonate (NaHCO_3_), urea, calcium chloride dihydrate (CaCl_2_·2H_2_O_2_), ammonium chloride (NH_4_Cl), dibasic sodium phosphate (Na_2_HPO_4_), monopotassium phosphate (KH_2_PO_4_), and magnesium chloride (MgCl_2_) were obtained from Desarrollo de Especialidades Quimicas, S.A. de C.V. (Monterrey, NL, Mexico). GIBCO (Carlsbad, CA, USA) supplied Dulbecco’s Modified Eagle Medium (DMEM), Penicillin–Streptomycin antibiotic (Pen-Strep), fetal bovine serum, phosphate-buffered saline (PBS, pH 7.4), antibiotic–antimycotic, and Hank’s buffered saline solution (HBSS). The Griess Reagent System and CellTiter 96 AQueous One Solution Cell Proliferation Assay Kits were purchased from Promega (G2930, Madison, WI, USA). Desulfoglucoraphanin was supplied by Santa Cruz Biotechnology (Dallas, TX, USA). IL-2, IL-6, IL-1β, and the TNF-α MILLIPLEX MAP Mouse Cytokine/Chemokine Panel and Human/Mouse Kit were obtained from Millipore (Billerica, MA, USA). The Total COX-2 DuoSet IC ELISA and Mouse IL-10 ELISA were purchased from R&D Systems (Minneapolis, MN, USA). The other chemicals were obtained from Sigma-Aldrich Co. (St. Louis, MO, USA)

### 2.2. Plant Material

Red Russian kale (*Brassica oleracea* var. acephala) seeds were supplied by La Semillería (Queretaro, Qro, Mexico). The kale seeds were disinfected with sodium hypochlorite (1.5%, *v*/*v*) for 15 min and then washed with Milli-Q water.

#### Selenium and Sulfur Treatments

The time and dose of the treatments were selected based on previously carried out studies [15]. Kale seeds were immersed in either Milli-Q water (control) or the treatment solutions for 5 h. Selenium (Se; 40 mg/L) and sulfur (S; 120 mg/L) were used as treatment solutions. After discarding the soaking solutions, the seeds were placed in germination containers for 7 days in a dark chamber set to 25 °C and 85% relative humidity. Concurrently, three replicates of each treatment were conducted. Each replicate consisted of a tray containing 30 g of kale seeds doused with 5 mL of different treatment solutions every 12 h for the duration of the experiment. After seven days of germination, samples were collected, freeze-dried (Labconco, Kansas City, MO, USA), pulverized into a powder, and stored at 80 °C until further phytochemical analysis.

### 2.3. Phytochemical Analyses

#### 2.3.1. Extraction of Secondary Metabolites

Based on the protocol published by Ortega-Hernández et al. [15], glucosinolates, phenolic compounds, and lutein were extracted in a single step from kale sprout powder (0.2 g) with 10 mL of pre-heated (70 °C) ethanol 70% (*v*/*v*) and spiked with 50 µL of sinigrin 3 mM as an internal standard.

The extracts were centrifuged (10,000 rpm, 10 min, 4 °C) (SL16R, Thermo Scientific, GER) to recover the supernatant after being allowed to cool at 25 °C. The resulting supernatant was utilized for further analysis.

#### 2.3.2. Identification and Quantification of Lutein and Phenolic Compounds

The ethanolic extracts were analyzed to identify and quantify lutein and phenolic compounds by HPLC-DAD (1260 Infinity, Agilent Technology, Santa Clara, CA, USA) using the conditions reported in our previous work [15].

#### 2.3.3. Desulfation of Glucosinolates

The ethanolic extracts (3 mL) were passed through polypropylene columns filled with 0.5 mL of DEAE-Sephadex A-25 resin pre-activated for at least 12 h in sodium acetate (0.02 M, pH 5). After eluting away the remaining supernatant, the columns were rinsed with 1 mL of water, followed by 1 mL of sodium acetate (0.02 M). Purified sulfatase (75 μL) was added to each column and was left at 25 °C overnight. Desulfoglucosinolates were diluted with 1.25 mL of water before HPLC analysis.

#### 2.3.4. Identification and Quantification of Glucosinolates

Glucosinolates were identified and quantified using an HPLC system (1260 Infinity, Agilent Technologies, Santa Clara, CA, USA) equipped with a diode array detector (DAD) set at 227 nm, using the same conditions reported in our previous work [15].

#### 2.3.5. Sulforaphane Analysis

The extraction and analysis of sulforaphane were carried out according to the method reported by González et al. [26], with slight modifications. Kale powder (1 g) was suspended in 10 mL of methylene chloride, combined with 0.5 g anhydrous sodium sulfate and 5 μL of butyl isothiocyanate (0.5 mg/mL) as an internal standard. After agitation for 60 min in a shaker in the absence of light, the sample was filtered, and the solution was vaporized at 30 °C under vacuum conditions (EZ-2.3, Genevac Ltd., Ipswich, UK) until dryness. For quantification, the residue was dissolved in 1 mL of ethanol and processed as described below.

Determination of sulforaphane was performed as reported by Torres-Contreras [27]. An HPLC-DAD (1260 Infinity, Agilent Technologies, Santa Clara, CA, USA) and a C18 reverse phase column (250 × 4.6 mm, 5 μm) (Luna, Phenomenex, Torrace, CA, USA) were used. The mobile phase consisted of water (phase A) and methanol (phase B). The gradient solvent system was 0/100, 10/90, 35/0, 40/0, and 50/100 (min/% phase A) at a flow rate of 0.8 mL/min with an injection volume of 20 μL. Data were obtained at 227 and 254 nm. For quantification, a standard curve of sulforaphane in the range of 0–100 ppm was generated, and results were expressed as mg per kg of kale (DW).

### 2.4. Selenium and Sulfur Determination

The chemical determination of the Se content in kale sprouts treated with Se, S, or water (control) was performed by inductively coupled plasma optical emission spectroscopy (ICP-OES) (Optima 4300 DV, PerkinElmer Instruments, Norwalk, CT, USA), using internal method INS-SM/US-71 based on EPA method 6010B. Sulfur content in kale was analyzed by inductively coupled plasma–atomic emission spectrometry (ICP-AES) (Optima 8300, PerkinElmer Instruments, Norwalk, CT, USA), using the Mexican codex NOM-117-SSA1-1994 according to EPA method 6010B.

### 2.5. Spray-Drying Encapsulation Process

To prepare the phytochemical extract, 18 g of Se- and S-treated kale sprouts were mixed with 200 mL of ethanol/water (70:30, *v*/*v*), pre-heated at 70 °C for 10 min to stop myrosinase activity. Then, samples were additionally heated in a water bath at 70 °C for 5 min and filtered to remove the fiber from the liquid. The supernatant was recovered.

Spray-drying was employed to prepare kale sprout powders according to the method reported by Radünz et al. [22]. Feeding solutions were prepared by mixing a maltodextrin solution (MD, 9% *w*/*v*) with kale sprout solutions, assaying three different ratios, 30:70, 50:50, and 70:30 *w*/*w* [23,25]. Microencapsulation was carried out in a Yamato Spray Dryer ADL311S (Yamato Scientific Co., Ltd., Tokyo, Japan) under the following operating conditions: inlet temperature of 120 °C, pump flow rate of 8.5 mL/min, outlet temperature of 60 °C, and pressure of 0.15 MPa. The operating conditions were selected based on preliminary tests. Each sample powder was stored in a dark plastic bag at 4 °C.

### 2.6. Encapsulation Efficiency

The encapsulation efficiency (EE) was determined by the glucosinolate, phenolic, and lutein content evaluated by HPLC-DAD [22]. For this, glucosinolates, lutein, and phenolics were quantified on kale extract microcapsules (0.25 g) according to the method described in Section 2.3. EE was expressed as a percentage and was calculated according to Equation (1).

(1) $$EE\left(\%\right)=\frac{Phytochemicals\;of\;kale-Phytochemicals\;of\;the\;capsule}{Phytochemicals\;of\;kale}$$

### 2.7. Storage Stability

The storage stability analysis was based on the procedure reported by Wu et al. [25]. Each treatment and wall proportion sample was stored at 35 °C for 28 days. Afterward, sampling for the quantification of glucosinolates, lutein, and phenolic compounds by HPLC-DAD (Section 2.3) was carried out. The preservation percentage of encapsulated and non-encapsulated phytochemicals after storage was used to evaluate their storage stability.

### 2.8. Scanning Electron Microscopy

Scanning electron microscopy (SEM) was carried out to characterize the microcapsule size and the uniformity of the microspheres, as well as to evaluate the surface morphology in dry experimental powder (maltodextrin 50:50 *w*/*w*). The microcapsule formulations were covered with gold 2.5 kV in the ionizer JEOL JFC-1100 and images were obtained at 6000× or 7000× magnification using SEM (JSM6360LV, Jeol) at the ICML, UNAM. The mean particle diameters were determined, ranging from 0.2 to 400 μm.

### 2.9. In Vitro Bioaccessibility of Kale Sprouts

To evaluate the bioaccessibility of the encapsulated and non-encapsulated phytochemicals present in kale sprouts, an *in vitro* study was conducted using the method reported by Flores et al. [28]. Briefly, 0.5 g of the sample was homogenized with 3 mL of simulated salivary fluid (FSS) for 2 min at 37 °C. Then, 6 mL of simulated gastrointestinal fluid (FGS) was added, and the pH was lowered to 2.0 ± 0.2. After sample incubation for 2 h at 37 °C, 6 mL of simulated intestinal fluid (FIS) and 3 mL of bile juices were added, the pH value was changed to 7.0 ± 0.2, and samples were incubated for another 2 h. The composition of the artificial digestive juices is presented in Table S1.

Aliquots were taken after each phase (oral, gastric, and intestinal) of the simulated digestion and subjected to 90 °C for 5 min for enzyme inactivation. After this, samples were centrifuged at 10,000 rpm for 10 min at 4 °C, and the resulting supernatants were stored at –80 °C until freeze-dried (Labconco, Kansas City, MO, USA). Samples were kept at −80 °C until the HPLC-DAD study of glucosinolate, lutein, and phenolic profiles using the protocol proposed in Section 2.3.

### 2.10. Cell Permeability of Phytochemicals in Digested Kale Sprouts

Human colon (Caco-2) cell monolayers were used to evaluate the *in vitro* cell permeability of lutein, glucosinolate, and phenolic compounds after the simulated gastrointestinal digestion of encapsulated and non-encapsulated kale sprouts treated with Se and S.

Caco-2 cells were cultured in a 37 °C and 5% CO_2_ atmosphere in Dulbecco’s Modified Eagle Medium (DMEM) containing 5% (*v*/*v*) fetal bovine serum and 0.5% antibiotic–antimycotic. The medium was replenished every two days during the cell growth and differentiation phases. For the assays, cells were inoculated at a density of 1 × 10^6^ cells/well in 12-well transwell inserts. The cell culture was used after 21 days, ensuring a confluent monolayer and complete cell differentiation [29]. Cells were washed twice with PBS. An aliquot of 0.5 mL of intestinal-digested fractions of encapsulated and non-encapsulated kale sprouts (120 µg/mL) containing lucifer yellow stain (LY) 100 µM was added to the upper chamber (apical side) and 1.5 mL of HBSS (pH 7.4) was added to the lower chamber (basolateral side). Cells were incubated at 37 °C for 2 h at 100 rpm.

After this, medium from both sides of the inserts was collected, treated to achieve dryness, and stored at −80 °C until HPLC-DAD analysis (Section 2.3). For chromatographic analysis, samples were reconstituted in 0.5 mL of ethanol/water (70:30, *v*/*v*).

The integrity of the cell monolayers at the end of the assay was assessed by testing lucifer yellow (LY) permeation. The fluorescence values of apical and basolateral samples were obtained using a microplate reader (Synergy HT, BioTek, Winooski, VM, USA) at 530 nm/485 nm (emission/excitation). The apical to basal LY permeation (%) was calculated according to Equation (2):

(2) $$LY\left(\%\right)=\frac{\left(F_{test}-F_{blank}\right)}{\left(F_{0}-F_{blank}\right)}$$

where F_test_ is the basolateral fluorescence intensity of LY; F_0_ is the initial apical fluorescence intensity of LY; F_blank_ is the fluorescence intensity of the blank sample (only HBSS). Only monolayers with less than 5% permeability were investigated for this experiment.

### 2.11. In Vitro Biological Activity of Kale Sprouts Subjected to Gastrointestinal Digestion

The effect of intestinal-digested fractions of encapsulated and non-encapsulated kale sprouts treated with Se and S on cellular antioxidant activity (CAA), nitric oxide (NOx), and cytokine (IL-1β, IL-2, IL-6, IL-10, TNF-α, and COX-2) production was evaluated.

#### 2.11.1. Cellular Antioxidant Activity (CAA)

To evaluate the cellular antioxidant activity of intestinal-digested fractions of encapsulated and non-encapsulated kale sprouts, the method described by Ortega-Hernández et al. [30] was used (Supplementary Materials).

#### 2.11.2. Nitric Oxide Determination

The production of nitric oxide was evaluated using the method described by Ortega-Hernández et al. [30] (Supplementary Materials).

#### 2.11.3. Measurement of COX-2, IL-1β, IL-2, IL-6, IL-10, and TNF-α

The effect of intestinal-digested fractions of encapsulated and non-encapsulated kale sprouts treated with Se and S on proinflammatory and anti-inflammatory cytokines was evaluated in Raw 264.7 cells. After the incubation of cells with the different treatments, the release of COX-2 and IL-10 was determined using the Human/Mouse Total COX-2 DuoSet IC and Mouse IL-10 ELISA Kits, per the respective manufacturer’s instructions. The absorbance values of cytokines were measured using a Synergy HT plate reader (Bio-Tek Instruments, Inc., Winooski, VT, USA) at 450 nm. Likewise, the MILLIPLEX MAP Mouse Cytokine/Chemokine Panel was used to measure IL-1β, IL-2, IL-6, and TNF-α in supernatants on a Luminex^R^ 200^TM^ System with the xPONENT@3.0 software (Luminex, TX, USA). From the immunoassay, Median Fluorescent Intensity (MFI) data using a polynomial curve-fitting method were used to calculate cytokine concentrations, as per the manufacturers’ guidelines.

### 2.12. Statistical Analysis

Statistical analyses were performed utilizing three replicates. The results were expressed as mean values and their standard error. Statistical analyses were conducted using the JMP version 13.0 software (SAS Institute Inc., Cary, NC, USA). Full factorial analyses of variance (ANOVA) followed by the least significant difference (LSD) test (*p* 0.05) were used to analyze the data.

## 3. Results and Discussion

### 3.1. Selenium and Sulfur Determination in Kale Sprouts

Treatment with Se during the germination of kale showed an 83.5-fold increase in its accumulation in sprouts after 7 days (21.13 mg/kg) compared to the untreated control (<0.25 mg/kg). Likewise, the kale sprouts germinated in the presence of S had a 1.125-fold increased concentration (3533 mg/kg) compared to the untreated control (3138 mg/kg). Selenium has chemical similarities to sulfur (S), and the sulfate transporters in root plasma membranes are anticipated to uptake selenate [31]. Despite the significant increment in Se in the treated sprouts, it did not negatively affect the growth and development of kale sprouts. Tian et al. [32] found that Se treatments harmed plant growth in the absence of S. Due to kale possessing an innately high S concentration, it is suggested that the nonspecific integration of Se into proteins decreased, compensating the redox system and preventing Se toxicity during kale biofortification.

### 3.2. Encapsulation Efficiency and Capsule Morphology

Encapsulation with maltodextrin at 50:50 showed the highest encapsulation efficiency (EE) (69.29–88.43%) (Table 1). This may have been the optimal concentration for non-saturation of the active maltodextrin sites, which permitted the total encapsulation of the combined extract, as well as additional protection of the compounds [22]. This result was superior to those reported by Tian et al. [24] and Wu et al. [25], who reported EE ranging from 12% to 77% for glucosinolate and isothiocyanate extracts derived from broccoli by spray-drying, employing maltodextrin. This difference in efficiency is possibly due to the coating materials, as maltodextrin exhibits greater efficacy for water-soluble compounds [33]. Radünz et al. [22] reported a similar EE for phenolic compounds in a Brassica vegetable of 97.8%, compared to the 90.3% achieved in our study. Whereas EE for lutein has not been reported, we found maximum efficiency with Se 50:50 of 40%. This low efficiency may be associated with the susceptibility of the polyene backbone of the lutein to the conditions present during encapsulation processing. Energy in the form of light, heat, and mechanical stress can interrupt the conjugation of the molecule, causing its fragmentation [34].

**Figure 1:**
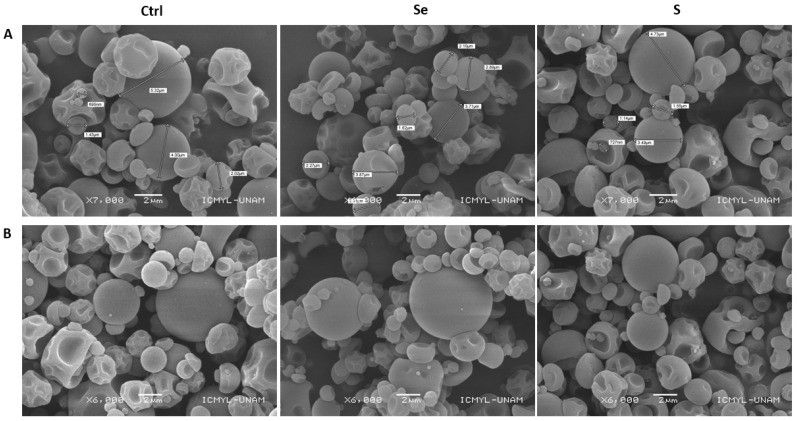
Electron microscopic images showing the morphology of encapsulated ethanolic extracts of 7-day-old Red Russian kale germinated with water (Ctrl), sulfur (S), and selenium (Se) generated by spray-drying at 7000× (**A**) and 6000× (**B**) magnifications. Extracts were encapsulated with maltodextrin in a ratio of 50:50.

The morphology of capsules with 50:50 *w*/*w* proportions of the kale sprout solutions and maltodextrin is shown in Figure 1. Capsules showed spherical structures and a smooth surface, confirming good encapsulation. The high drying rate and rapid solidification of the maltodextrin wall could have caused the buckling. Previous studies that used maltodextrin as an encapsulating material for broccoli extracts by spray-drying reported that they obtained an irregular and rough surface due to the droplets’ high temperature and rapid evaporation [24,25].

The particle size was in the range of 2–6 μm, which can be considered a fine particle size that provides better techno-functional properties to generate food and pharmaceutical products.

### 3.3. Influence of Encapsulation and Storage on Lutein, Phenolic, and Glucosinolate Compounds

**Figure 2:**
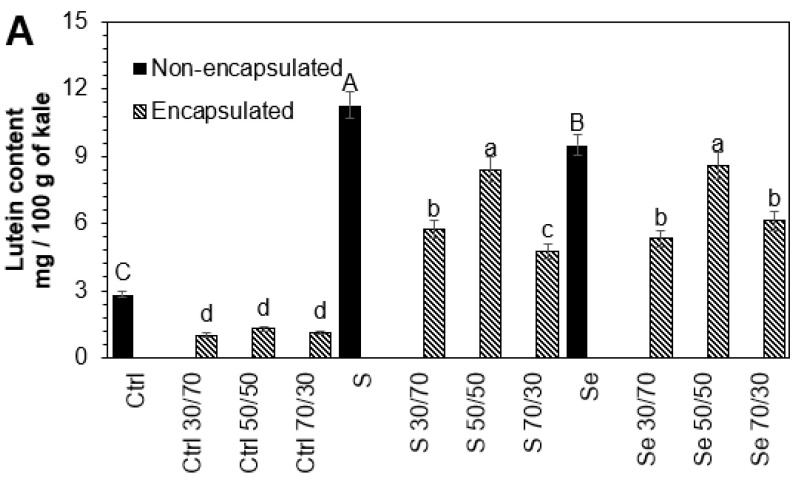

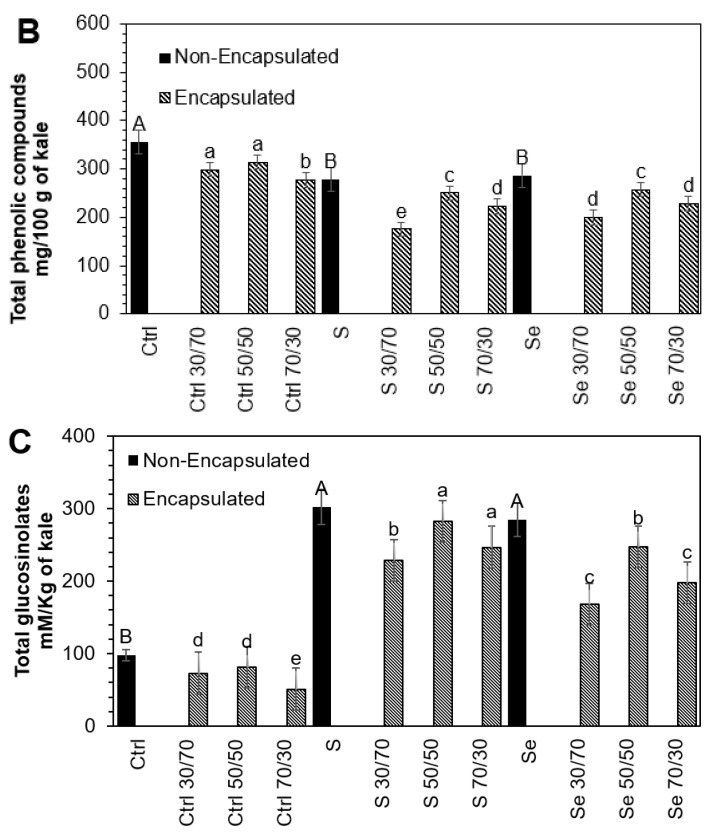
The concentrations of (**A**) lutein, (**B**) total phenolic compounds, and (**C**) total glucosinolates in encapsulated and non-encapsulated extracts of 7-day-old Red Russian kale sprouts germinated with water (Ctrl), sulfur (S), and selenium (Se). Extracts were encapsulated with maltodextrin in ratios of 30:70, 50:50, and 70:30 *w*/*w*. Bars represent the means of 3 replicates ± standard error. Different letters indicate a statistically significant difference between all treatments, as determined by Tukey’s HSD test (*p* < 0.05). Abbreviations: control (Ctrl), sulfur (S), selenium (Se).

The stability of phytochemicals during the encapsulation process depends on the interaction within the core-to-wall ratio (Figure 2). An intermediate core (50:50 *v*/*v*) ratio resulted in the higher stability of phytochemicals in all tested samples, followed by the 30:70 and 70:30 core-to-wall ratios. The lutein (54.2%, 25.4%, 9.6%), phenolic compounds (13.5%, 10.1%, 11.4%), and glucosinolates (16.9%, 6.4%, 13.1%) present in kale seeds germinated with water, sulfur, and selenium, respectively, suffered a small but significant loss during the encapsulation process with maltodextrin 50:50 *w*/*w* compared to non-encapsulated samples. Maltodextrin has been shown to preserve the carotenoids in paprika oleoresin and to offer adequate oxidative stability for encapsulated olive oil [35,36]. Likewise, it has been found that maltodextrin is an effective protector of phenolic extracts and can preserve their antioxidant activity [37]. The degradation of phytochemicals during the encapsulation process is mainly due to direct exposure to heat [25].

**Figure 3:**
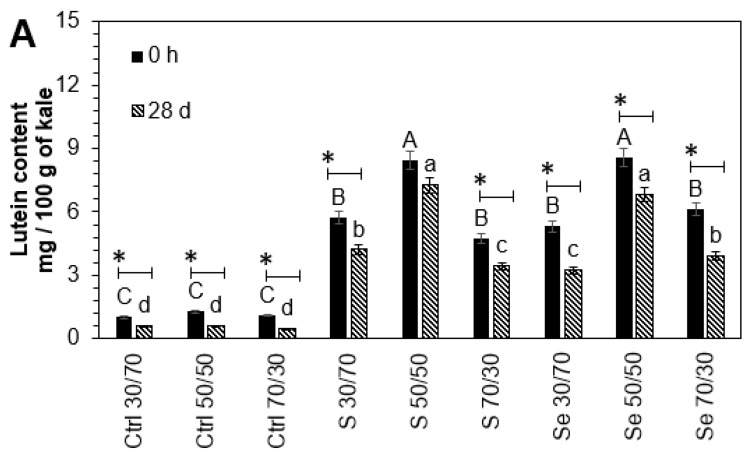

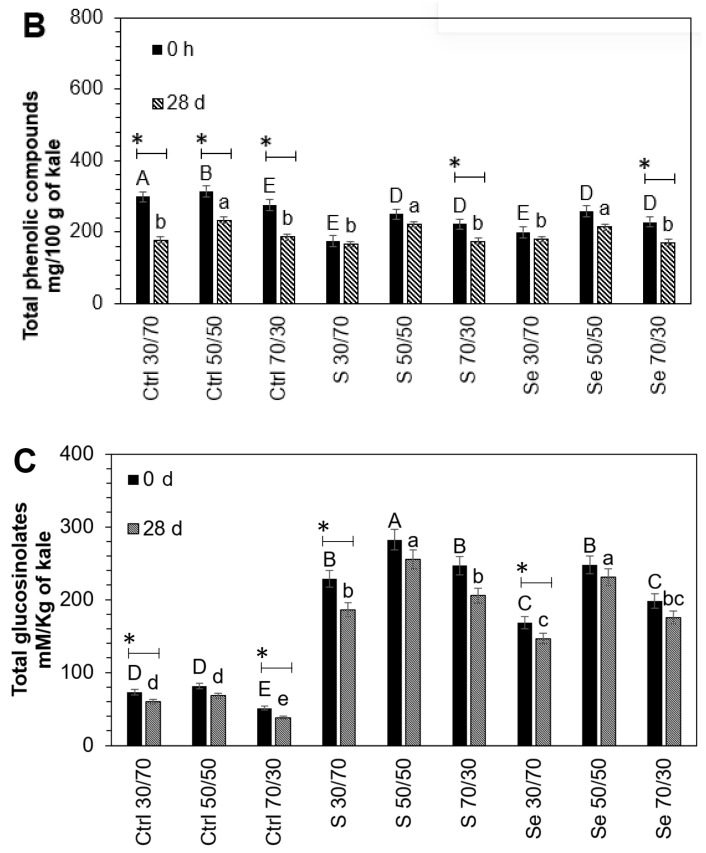
The concentrations of (**A**) lutein, (**B**) total phenolic compounds, and (**C**) total glucosinolates in encapsulated extracts of 7-day-old Red Russian kale sprouts germinated with water (Ctrl), sulfur (S), and selenium (Se) before (0 days) and after storage (28 days). Extracts were encapsulated with maltodextrin in ratios of 30:70, 50:50, and 70:30. Bars represent the means of 3 replicates ± standard error. Different letters indicate a statistically significant difference between all treatments, as determined by Tukey’s HSD test (*p* < 0.05). Asterisk (*) indicates the statistical difference determined by a *t*-test (*p* < 0.050) between non-stored (0 h) and stored (28 days) encapsulated kale samples. Abbreviations: control (Ctrl), sulfur (S), selenium (Se).

After storage, there was an additional loss in phytochemicals; however, the 50:50 maltodextrin encapsulation treatment remained optimal for their protection. The results are shown in Figure 3. The lutein (56.6%, 27.1%, 36.9%), phenolic compounds (32.5%, 21.2%, 25%), and glucosinolates (25.5%, 16.1%, 12.4%) present in encapsulated (70:30 *w*/*w*) control and treated kale sprouts with sulfur and selenium, respectively, suffered a higher loss after the storage of capsules compared to non-encapsulated samples. These storage stability results may be explained based on the fact that maltodextrin can create a dense and oxygen-impermeable wall system. This effect has been reported previously by Castro-Enríquez et al. [38], who observed better storage stability for betacyanins using maltodextrin as an encapsulant after 16 weeks. In this way, treatment with a 30:70 ratio, where phytochemicals were near the outer surface of the capsule, led to a greater loss. Since the wall was thinner than the core, the phytochemicals could react with the outside oxygen.

### 3.4. Bioaccessibility of Lutein, Phenolic, and Glucosinolate Compounds

*In vitro* stimulation of gastrointestinal digestion provides important information on how the chosen compounds behave in a physiological environment [19].

The first step in the absorption process is the release of phytochemicals from the food matrix. The liberation of lutein after simulated saliva solubilization and the *in vitro* digestion of non-encapsulated and encapsulated kale sprout extracts is presented in Table S2.

The liberation of lutein from the non-encapsulated and encapsulated kale sprout extracts treated with S and Se was significantly increased, through the mouth (24.2%, 35.2%), stomach (31.7%, 40.6%), and intestine (40.1%, 45.5%) fractions, compared with the concentrations of raw kale sprouts, respectively. These results are in line with Eriksen et al. [39], who reported 30% carotenoid release from pureed spinach when studying *in vitro* accessibility. The differences in carotenoid release could be influenced by factors such as domestic processing and the specific matrix. Moreover, only encapsulated extracts treated with Se presented the significant liberation of lutein, in contrast with the non-encapsulated samples, during *in vitro* digestion: mouth 23.9%, stomach 19.7%, and intestine 22.6%.

Comparing the concentrations of phenolic compounds in the raw samples of the encapsulated and non-encapsulated kale sprouts with the mouth, stomach, and intestine fractions of digestion *in vitro*, all eight identified compounds were significantly lost at all stages. The observed reductions in phenolic compounds are available in Table S3. The greatest reduction at the intestinal level was observed in the non-encapsulated control samples: 4-O-caffeoylquinic acid (87%), 3-O-hexoside kaempferol (89%), sinapic acid (89%), ferulic acid (88%), 1-sinapoyl-2′-ferulolylgentiobiose (94%), 1,2-disinapoyl-2-ferulolylgentiobiose (90%), kaempferol 3-*O*-sophoroside-7O-glucoside (90%), quercetin (90%). These results are in agreement with what was reported by Oidtmannet al. [40], Betzet al. [41], and Flores et al. [28], who noted a decrease ranging between 64% and 78% in total phenolics in indigestion conditions. Differences in carotenoid degradation may be associated with the product-related protein–polyphenol interactions in each matrix, and the process-related heating step used for encapsulation. However, there is currently a lack of data in the literature on the estimation of phenolics in encapsulated kale extracts after *in vitro* digestion.

Losses observed for nearly all phenolic compounds analyzed may have resulted from structural disintegration caused by the action of digestive enzymes and pH fluctuations during the digestive process. Moreover, the high affinity of phenolics to maltodextrin through non-covalent binding, including interactions between hydrophobic groups, may stabilize the polyphenol structure [42].

On the other hand, the encapsulated kale sprouts treated with Se had better phenolic compound stability than non-encapsulated kale sprouts during *in vitro* digestion. At the intestinal level, the additional reductions in phenolic compound content in non-encapsulated kale sprouts compared to encapsulated samples treated with Se were as follows: 4-O-caffeoylquinic acid (30%), 3-O-hexoside kaempferol (18%), sinapic acid (26%), ferulic acid (15%), 1-sinapoyl-2′-ferulolylgentiobiose (20%), 1,2-disinapoyl-2-ferulolylgentiobiose (22%), kaempferol 3-*O*-sophoroside-7O-glucoside (43%), quercetin (33%).

Furthermore, individual glucosinolates were identified and quantified in raw kale sprouts. The observed reductions in glucosinolates are available in Table S4. The greatest reduction at the intestinal level was observed in the non-encapsulated control kale sprouts: glucoiberin (100%), progoitrin (100%), glucoraphanin (50%), gluconapin (100%), 4-hydroxy-glucobrassicin (100%), glucoeurocin (100%), glucobrassicin (60%), 4-methoxy-glucobrassicin (75%). Similarly, reductions in glucosinolate compounds in all samples were found during *in vitro* digestion. Changes in concentration may be due to restructuring with other compounds, such as sulforaphane. Once digestion begins, glucosinolates undergo enzymatic hydrolysis under the action of myrosinase and are hydrolyzed to isothiocyanate [43].

On the other hand, the S- and Se-encapsulated kale sprouts showed higher stability than non-encapsulated samples at the intestinal level for progoitrin (29%, 35%), gluconapin (32%, 27%), and 4-methoxy-glucobrassicin (10%, 13%), respectively. Thus, *in vitro* digestion proved that encapsulation is a suitable method to improve the phytochemical stability of kale, leading to less reduction as well as better bioaccessibility.

### 3.5. Cellular Permeability

The integrity of the cell monolayers was assessed by monitoring LY permeation. Only monolayers with LY permeability lower than 5% were considered for the experiment. The data obtained are presented in Table 2.

After 2 h, the phytochemical compounds in the apical and basolateral compartments were analyzed. In the case of glucosinolate quantification, both glucosinolates and the hydrolysis products were present in the intestinal digesta at 2 h. However, only the hydrolysis products were analyzed in the Caco-2 cells because, as expected, intact glucosinolates were not absorbed through the intestinal epithelial cells.

HPLC-DAD analysis of the polyphenols permeating to the basolateral side after 120 min revealed the presence of five identified compounds: lutein, sulforaphane, ferulic acid, 3-O-hexoside kaempferol, and quercetin.

The highest basolateral recovery of phytochemicals was found in Se-encapsulated kale sprouts. Lutein, sulforaphane, ferulic acid, 3-O-hexoside kaempferol, and quercetin recovery ranged between 8.4% and 67.4%. This result agrees with previous *in vitro* tests on the permeation of encapsulated phytochemicals across Caco-2 cells [44,45,46]. Faralli et al. [45] reported a direct effect of the encapsulant on the tight junctions and, thus, an increase in the permeation of curcumin through the epithelial barrier. Panwar et al. [47] found that encapsulated ferulic acid showed higher plasma retention, compared with ferulic acid in free form, and thus provided greater bioavailability. Even phytochemicals undergo enzymatic degradation during absorption across intestinal epithelial cells [48]. Therefore, encapsulated phytochemicals enabled resistance in an acidic environment and complete release in alkaline conditions, thus enhancing the permeability.

In addition, the presence of two unidentified peaks, tentatively attributed to ferulic acid, in the basolateral compartment and not in the initial sample suggests that it resulted from cell metabolism. Kern et al. [49] reported that Caco-2 cells can absorb ferulic acid, p-coumaric acid, and sinapic acid and conjugate them by sulfation or glucuronidation before excretion.

### 3.6. Cellular Antioxidant Activity and Nitric Oxide Production

**Figure 4:**
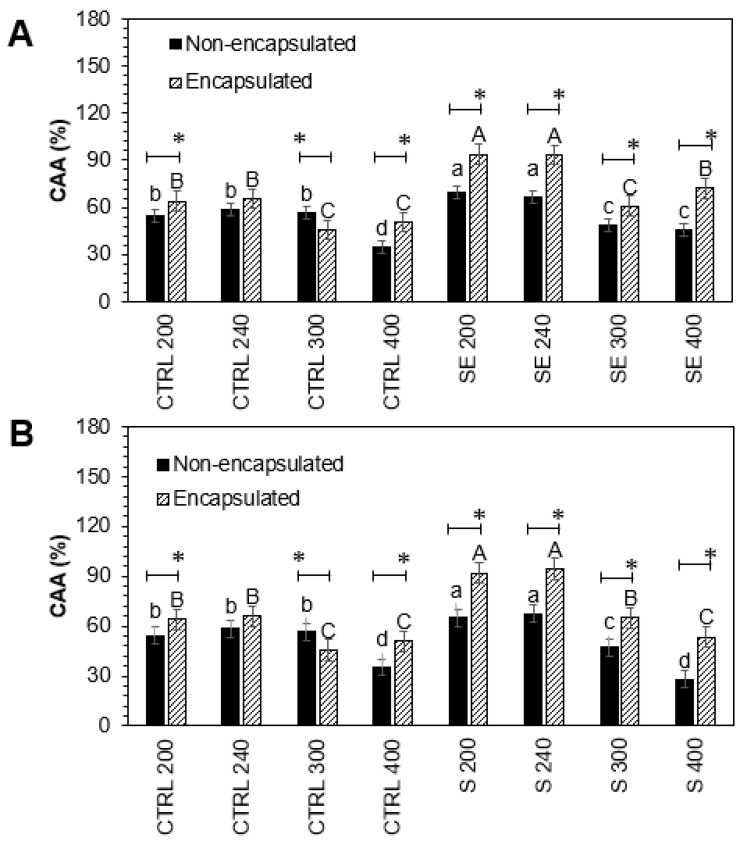
Cellular antioxidant activity of encapsulated and non-encapsulated intestinal-digested fractions (200, 240, 300, and 400 µg/mL) of Red Russian kale sprouts treated with (**A**) selenium and (**B**) sulfur after 7 days of germination in Caco-2 cells. Bars represent the means of 3 replicates ± standard error. Different letters indicate a statistically significant difference between all treatments, as determined by Tukey’s HSD test (*p* < 0.05). Asterisk (*) indicates statistical difference determined by a *t*-test (*p* < 0.050) between encapsulated and non-encapsulated kale pair samples. Abbreviations: control (Ctrl), sulfur (S), selenium (Se).

As is known, many biologically active molecules in plants may contribute to the antioxidant capacity, and kale is not an exception. The cellular antioxidant activity of encapsulated and non-encapsulated intestinal-digested fractions of Red Russian kale sprouts treated with Se and S in Caco-2 cells is shown in Figure 4.

There was a significant increase in cellular antioxidant activity in non-encapsulated and encapsulated intestinal-digested fractions treated with S 200 (19.7%, 43.7%), S 240 (15.5%, 43.7%), Se 200 (28.9%, 46.5%), and Se 240 (13.8%, 42.3%), compared to controls, respectively.

Although the Se and S treatments were not significantly different, the encapsulation process positively affected the cellular antioxidant activity. Encapsulated intestinal-digested fractions S 200 (23.9%), S 240 (28.2%), Se 200 (17.6%), and Se 240 (28.4%) showed a significant increase compared with their respective non-encapsulated pairs.

**Figure 5:**
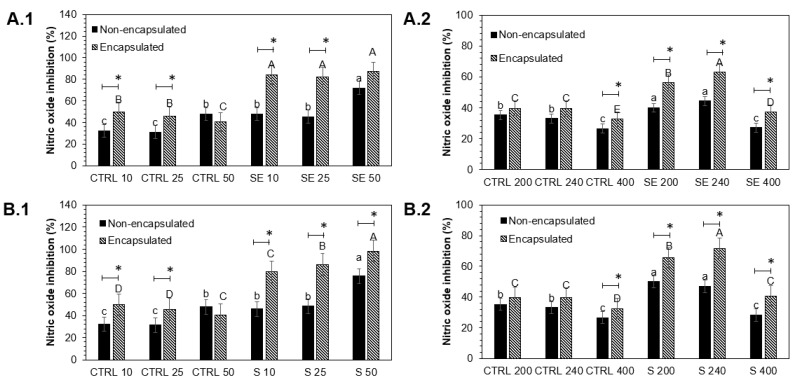
Effect of encapsulated and non-encapsulated intestinal-digested fractions of Red Russian kale sprouts treated with (**A**) selenium and (**B**) sulfur after 7 days of germination on nitric oxide production by macrophage (**1**) Raw 264.7 (10, 25, and 50 µg/mL) and (**2**) Caco-2 cells (200, 240, and 400 µg/mL) stimulated with 1 μg/mL lipopolysaccharide. Bars represent the means of 3 replicates ± standard error. Different letters indicate a statistically significant difference between all treatments, as determined by Tukey’s HSD test (*p* < 0.05). Asterisk (*) indicates statistical difference determined by a *t*-test (*p* < 0.050) between encapsulated and non-encapsulated kale pair samples. Abbreviations: control (Ctrl), sulfur (S), selenium (Se).

The anti-inflammatory activity of encapsulated and non-encapsulated intestinal-digested fractions of Red Russian kale sprouts treated with Se and S in Raw 264.7 and Caco-2 cells is shown in Figure 5.

Raw 264.7 cells showed a significant increase in nitric oxide inhibition in non-encapsulated and encapsulated samples treated with all S and Se extract concentrations tested. The maximum nitric oxide inhibition was observed with non-encapsulated and encapsulated S 50 (51.1%, 115.2%) and Se 50 (58.6%, 143.1%) intestinal-digested fractions compared to controls, respectively. On the other hand, the highest increase in nitric oxide inhibition in Caco-2 cells was detected with non-encapsulated and encapsulated intestinal-digested fractions S 240 (42.1%, 80.9%) and Se 240 (34.3%, 58.8%), followed by S 200 (41.6%, 64.9%) and Se 200 (13.3%, 41.5%), compared to controls, respectively.

Although the Se and S treatments were not significantly different in Raw 264.7 cells, the encapsulation process positively affected the cellular antioxidant activity. Encapsulated samples S 50 (64.1%) and Se 50 (84.3%) showed a significant increase compared with their respective non-encapsulated pairs. In the case of Caco-2 cells, encapsulation showed a significant increase in S 200 (23.2%), S 240 (38.9%), Se 200 (28.2%), and Se 240 (24.5%) samples.

These results indicate that the antioxidant and anti-inflammatory activity of the Se and S tested sprouts, evaluated by the cellular method, was positively correlated with the release of lutein and sulforaphane in the extracts, as well as the remaining content of phenolic and glucosinolate compounds, which was higher than in control samples. The influence of lutein, glucosinolates, and phenolic compounds in neutralizing free radicals, quenching singlet oxygen, or decomposing peroxides through their chemical structure has been reported previously [50,51,52], whereas the antioxidant activity of sulforaphane is mainly associated with the transcription triggering of phase II metabolic enzymes [53]. Additionally, the antioxidant properties of phytochemical extracts in S- and Se-enriched Brassica plants such as broccoli [54], kale [15,55], and garden cress [56] have been confirmed.

### 3.7. Measurement of COX-2, IL-1β, IL-2, IL-6, IL-10, and TNF-α

**Figure 6:**
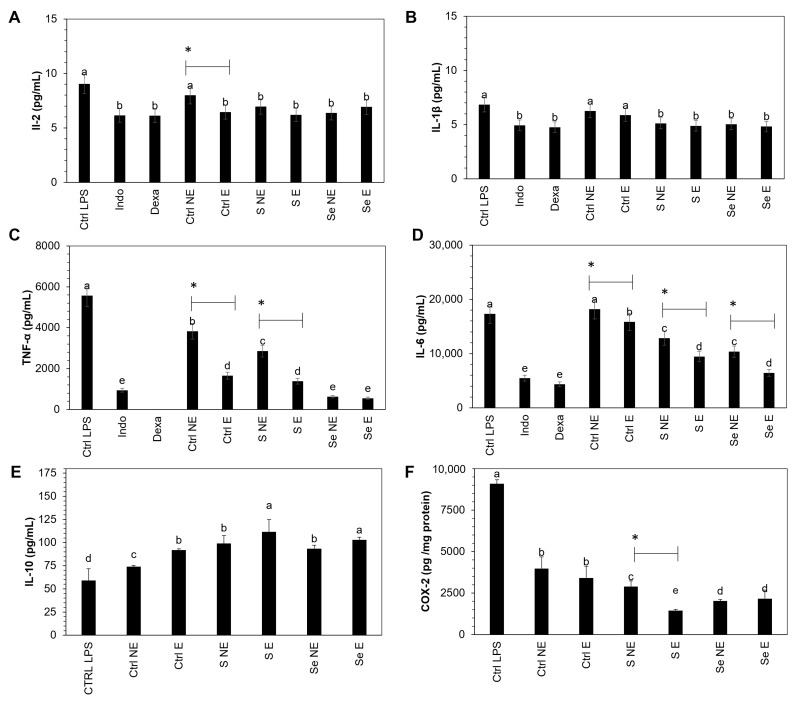
Effect of encapsulated and non-encapsulated intestinal-digested fractions (50 µg/mL) of Red Russian kale sprouts treated with selenium and sulfur after 7 days of germination on (**A**) IL-2, (**B**) IL-1β, (**C**) TNF-α, (**D**) IL-6, (**E**) IL-10, and (**F**) COX-2 production by macrophage Raw 264.7 cells stimulated with 1 μg/mL lipopolysaccharide. Bars represent the means of 3 replicates ± standard error. Different letters indicate a statistically significant difference between all treatments, as determined by Tukey’s HSD test (*p* < 0.05). Asterisk (*) indicates a statistical difference between encapsulated and non-encapsulated kale pair samples. Abbreviations: control (Ctrl), sulfur (S), selenium (Se), lipopolysaccharide (LPS), encapsulated (E), non-encapsulated (NE), indomethacin (Indo), and dexamethasone (Dexa).

The influence of the S and Se kale sprout extracts on the release of the selected proinflammatory mediators (COX-2, IL-1β, IL-2, IL-6, IL-10, and TNF-α) in LPS-stimulated Raw 264.7 macrophages was confirmed as the final stage of this investigation. Results are shown in Figure 6.

Macrophages treated with kale extract showed an inhibition in the production of COX-2, IL-1β, IL-2, IL-6, and TNF-α by LPS-stimulated Raw 264.7 cells with the same potency as dexamethasone and indomethacin, two anti-inflammatory agents used as reference drugs, on the inhibition of cytokines.

Raw 264.7 cells showed significant inhibition in proinflammatory cytokines in non-encapsulated and encapsulated kale sprout samples, including the control, whereas IL-10 was stimulated. There was a significant increase of 24.1% and 13.9% in IL-10 in S- and Se-encapsulated kale samples compared to the non-encapsulated samples. COX-2 and TNF-α showed significant inhibition of 50.6% and 51.4% in S-encapsulated kale sprouts compared to non-S-encapsulated kale sprouts, respectively, whereas significant inhibition of 21.2% and 32.5% was observed in IL-6 in S- and Se-encapsulated kale samples, respectively.

Furthermore, the maximum inhibition was observed with S- and Se-encapsulated kale samples in the corresponding COX-2 (84.1%, 85.7%), IL-1β (28.5%, 29.6%), IL-2 (31.5%, 23.4%), IL-6 (36.6%, 54.3%), and TNF-α (75.1%, 90.1%) cytokines, respectively, compared to controls stimulated with LPS. In contrast, the IL-10 concentration increased significantly by 88.9% and 74.6% in S- and Se-encapsulated kale samples, respectively.

First, the observed tendency to decrease the anti-inflammatory cytokines may be explained by the capacity of the present phenolics and lutein to suppress the iNOS protein by inhibiting nuclear factor-κB (NF-κB), which is the key transcription factor for the activation of genes associated with the inflammatory mediators, such as interleukins, inducible nitric oxide synthase (iNOS), and cyclooxygenases (COXs) [57,58]. Additionally, the chemical structure and lutein and phenolic compounds allow them to scavenge NO radicals [2,2,59]. The potential of lutein, kaempferol, ferulic acid, and quercetin as anti-inflammatory agents has been reported previously [58].

On the other hand, lutein and sulforaphane can activate the nuclear factor erythroid 2-related factor 2 (Nrf2) and Nrf2-signaling-related antioxidant enzymes (glutathione-s-transferase, glutathione peroxidase, superoxide dismutase, and catalase) [60].

Moreover, the increment in IL-10, an anti-inflammatory cytokine, may inhibit the nuclear factor kappa light chain enhancer of activated B cells (NF-kB) translocation [61] and further production of the proinflammatory cytokines.

Finally, maltodextrin may protect the compounds causing the inhibition of free radicals during digestion, demonstrating that encapsulation is an effective method of preserving the antioxidant and anti-inflammatory properties of kale.

## 4. Conclusions

In this study, the carotenoid, glucosinolate, and phenolic profiles of kale sprouts (*Brassica oleracea* var. acephala) were characterized by HPLC-DAD. Additionally, an *in vitro* gastrointestinal method was used to determine the digestive stability and bioaccessibility of the most important antioxidants. We reported a total of 17 antioxidants composed of eight phenolic acids (sinapic acid, ferulic acid, 4-O-caffeoylquinic acid, 1-sinapoyl-2′-ferulolylgentiobiose, 3-O-hexoside kaempferol, 1,2-disinapoyl-2-ferulolylgentiobiose, kaempferol 3-O-sophoroside-7O-glucoside, and quercetin), eight glucosinolates (glucoiberin, progoitrin, glucoraphanin, gluconapin, 4-hydroxy-glucobrassicin, glucoeurocin, glucobrassicin, and 4-methoxy-glucobrassicin), and one carotenoid (lutein). It was found that these phytochemicals were more abundant in kale treated with Se and S, where they presented high bioaccessibility and bioactivity.

Furthermore, encapsulation protected carotenoids, phenolics, and glucosinolates from degradation during simulated digestion and storage in comparison with non-encapsulated phytochemicals, although the minor loss of encapsulated kale phytochemicals during *in vitro* digestion did not affect the permeability through the Caco-2 monolayer. Moreover, the encapsulated phytochemicals demonstrated better antioxidant and anti-inflammatory effects *in vitro* in Caco-2 and Raw 264.7 cells. This information was correlated with the positive modulation of mediators of inflammation (IL-2, IL-1β, TNF-α, IL-6, IL-10, and COX-2).

Therefore, this kale-based powder could be a functional ingredient candidate for the development of foods or supplements to prevent the development of diseases with a marked inflammatory component, or as an adjunct with pharmacological therapy to mitigate symptoms and improve the quality of life of patients. However, to better comprehend the potential effects of kale extracts on human health, additional research should be considered to evaluate the roles of these bioactive compounds in the gastrointestinal microbiota, including *in vivo* studies.

Finally, this study demonstrated that kale represents a hyperaccumulator model of Se without negatively affecting its growth or development. Therefore, it can be used to create biofortified foods of superior nutritional value, enriched with healthful Se compounds and several other valuable phytochemicals.

## Figures and Tables

**Table 1:** Encapsulation efficiency based on the phytochemical content.

Formulation	Glucosinolates (mM/kg) ^1^				Phenolics (mg/100 g) ^2^				Lutein (mg/100 g) ^2^			
CTRL 30:70	52.48	±	4.22	d	36.12	±	3.51	e	11.23	±	3.51	d
CTRL 50:50	69.29	±	3.43	c	45.72	±	4.42	d	17.81	±	4.42	c
CTRL 70:30	57.5	±	5.09	d	39.05	±	2.43	e	21.25	±	2.43	b
S 30:70	66.28	±	5.32	c	68.33	±	4.79	bc	16.43	±	4.79	d
S 50:50	85.97	±	7.63	a	74.54	±	6.35	b	21.25	±	6.35	c
S 70:30	61.23	±	4.56	c	59.61	±	4.38	c	25.00	±	4.38	b
SE 30:70	58.27	±	4.76	d	77.11	±	5.32	b	25.45	±	5.32	b
SE 50:50	88.43	±	6.43	a	90.37	±	7.53	a	40.35	±	7.53	a
SE 70:30	75.01	±	6.23	b	85.60	±	2.40	a	32.55	±	2.40	b

^1^ Results are expressed as mmol of desulfoglucoraphanin equivalents per kg of sample on dry weight basis. ^2^ Concentrations of lutein and phenolic compounds are expressed as mg of phytochemical per 100 g of sample (mg/100 g) on dry weight basis. Values represent the means of 3 replicates ± standard error. ^a, b, c, d, e^ Different letters indicate a statistically significant difference between all treatments, as determined by Tukey’s HSD test (*p* < 0.05).

**Table 2:** Cellular permeability after 2 h of digested encapsulated and non-encapsulated 7-day-old Red Russian kale sprouts treated with selenium and sulfur in Caco-2 cells.

	Concentration of Phytochemicals (mg/100 g) DW ^1,2^																								
Non-Encapsulated	Lutein					Sulforaphane					Ferulic Acid					3-O-H-K					Quercetin				
**Ctrl**	1.2	±	0.1	^c^	(22.4%)	3.7	±	0.4	^d^	(17.9%)	1.9	±	0.2	^d^	(10.4%)	1.1	±	0.1	^d^	(9.4%)	0.7	±	0.1	^d^	(8.4%)
**S**	1.3	±	1.2	^c^	(25.2%)	24.9	±	2.4	^c^	(34.2%)	4.5	±	0.4	^d^	(16.4%)	4.1	±	0.4	^c^	(14.8%)	1.8	±	0.2	^d^	(13.3%)
**Se**	1.6	±	0.4	^c^	(27.4%)	52.6	±	5.1	^b^	(36.3%)	11.9	±	1.2	^b^	(25.5%)	6.2	±	0.6	^b^	(23.0%)	2.6	±	0.3	^c^	(20.7%)
**Encapsulated**																									
**Ctrl**	1.1	±	0.1	^c^	(20.2%)	3.9	±	0.4	^d^	(19.2%)	9.3	±	0.9	^c^	(38.4%)	5.8	±	0.6	^c^	(34.6%)	2.9	±	0.3	^c^	(31.1%)
**S**	8.7	±	0.5	^b^	(45.0%)	59.5	±	5.8	^a^	(46.3%)	13.8	±	1.3	^b^	(43.3%)	7.8	±	0.8	^b^	(39.0%)	14.8	±	1.4	^a^	(35.1%)
**Se**	10.0	±	0.2	^a^	(57.4%)	66.0	±	6.4	^a^	(67.4%)	19.3	±	1.9	^a^	(54.5%)	9.8	±	1.0	^a^	(49.1%)	10.2	±	1.0	^b^	(44.1%)

^1^ Percentage values represent the proportion of recovery of each compound on the basal surface with respect to the initial concentration. ^2^ Different letters in the same column denote statistically significant differences in the concentration of each compound between treatments, as determined by the least significant difference (LSD) test (*p* < 0.05). Abbreviations: 3-O-hexoside kaempferol (3-O-H-K), non-encapsulated (NE), encapsulated (E), selenium (S), sulfur (S), and dry weight (DW).

## Data Availability

Data is contained within the article.

## Supplementary Materials

The following supporting information can be downloaded at: https://www.mdpi.com/article/10.3390/foods12112149/s1, Table S1: Composition of artificial digestive juices; Table S2: Bioaccessibility of lutein in encapsulated and non-encapsulated 7-day-old Red Russian kale sprouts treated with selenium and sulfur; Table S3: Bioaccessibility of individual phenolic compounds in encapsulated and non-encapsulated 7-day-old Red Russian kale sprouts treated with selenium and sulfur; Table S4: Bioaccessibility of individual glucosinolates in encapsulated and non-encapsulated 7-day-old Red Russian kale sprouts treated with selenium and sulfur; Methodology S2.11.1. Cellular antioxidant activity (CAA); Methodology S2.11.2. Nitric oxide determination.
