# Supplementary material for: Immunomodulatory and Antioxidant Effects of Spray-Dried Encapsulated Kale Sprouts after In Vitro Gastrointestinal Digestion
Source: Foods. 2023 May 26;12(11):2149. doi: 10.3390/foods12112149 (PMC10253027; doi:10.3390/foods12112149)
Supplement: Supplementary file 1 [file foods-12-02149-s001.zip › foods-2391017-supplementary.pdf]

Immunomodulatory and antioxidant effects of spray-dried encapsulated kale sprouts after *in vitro* gastrointestinal digestion

Erika Ortega-Hernández[1,2], Ana Victoria Camero-Maldonado[3], Laura Acevedo-Pacheco[1], Daniel A. Jacobo-Velázquez[4,5]* and Marilena Antunes-Ricardo[1,2]*

1 Tecnologico de Monterrey, Escuela de Ingeniería y Ciencias, Centro de Biotecnología-FEMSA, Av. Eugenio Garza Sada 2501 Sur, 64849, Monterrey, N.L., México.
2 Tecnologico de Monterrey, The Institute for Obesity Research, Ave. Eugenio Garza Sada 2501 Sur, 64849, Monterrey, N.L., México.
3 Tecnologico de Monterrey, Escuela de Medicina y Ciencias de la Salud, Av. Ignacio Morones Prieto 3000, 64710, Monterrey, N.L., México.
4 Tecnologico de Monterrey, Escuela de Ingeniería y Ciencias, Ave. General Ramón Corona 2514, 45201, Zapopan, Jal, México.
5 Tecnologico de Monterrey, The Institute for Obesity Research, Ave. General Ramón Corona 2514, 45201, Zapopan, Jal, México.
* Correspondence: marilena.antunes@tec.mx (M.A.R.); djacobov@tec.mx (D.A.J.-V)

Table S1. Composition of artificial digestive juices

Compound	Artificial saliva	Gastric juice	Intestinal juice	Bile juice
Distilled water	500 mL	500 mL	500 mL	500 mL
NaCl	58.5 mg	2.752 g	7.012 g	5.259 g
KCl	74.5 mg	0.824 g	0.564 g	0.375 g
$NaHCO_3$	1.05 g		3.388 g	5.785 g
Urea	0.2 g	0.085 g	0.1 g	0.25 g
Na_2HPO_4		0.266 g		
$CaCl_2$*$2H_2O_2$		0.399 g		
NH_4Cl		0.306 g		
KH_2PO_4			80 mg	
$MgCl_2$			50 mg	
α-amylase	1.0 g			
Pepsin		2.5 g		
Pancreatin			9 g	
Lipase			1.5 g	
Bile salts				30 g
pH	7.0 ± 0.2	2.0 ± 0.2	7.0 ± 0.2	7.0 ± 0.2

Table S2. Bioaccessibility of lutein in encapsulated and non-encapsulated 7-day-old Red Russian kale sprouts treated with selenium and sulfur.

	Non-Encapsulated			Encapsulated		
	**Ctrl**	**S**	**Se**	**Ctrl**	**S**	**Se**
**Raw kale**	2.8[1] ± 0.1 b[2]	11.3 ± 1.2 c	9.5 ± 0.4 c	1.3 ± 0.1 b	8.4 ± 0.5 b	8.57 ± 0.2 b
**Mouth**	2.9 ± 0.2 b 3.40%[3]	12.85 ± 1.4 b 12.10%	10.8 ± 1.1 b 11.70%	1.4 ± 0.1 b 5.90%	11.1 ± 0.7 b 24.20%	13.3 ± 0.4 a 35.20%
**Stomach**	3.7 ± 0.1 a 23.40%	14.74 ± 1.7 a 23.20%	12 ± 1.5 a 20.90%	1.6 ± 0.1 a 20.90%	12.3 ± 1.6 a 31.70%	14.4 ± 1.3 a 40.60%
**Intestine**	4.1 ± 0.1 a 31.40%	16.67 ± 1.2 a 32.40%	12.4 ± 1.5 a 23.10%	1.8 ± 0.1 a 28.30%	14.2 ± 1.4 a 40.10%	15.9 ± 1.5 a 45.40%

[1] Concentration is reported mg per 100 g of kale (DW). [2] Different letters in the same column indicate statistical differences in the concentration of each compound between treatments using the least significant difference (LSD) test ($p < 0.05$). [3] Percentage values represent the proportion of liberation of lutein with respect to the control. Abbreviations: non-encapsulated (NE), encapsulated (E), selenium (S), and sulfur (S).

Table S3. Bioaccessibility of individual phenolic compounds in encapsulated and non-encapsulated 7-day-old Red Russian kale sprouts treated with selenium and sulfur.

	Non-Encapsulated						Encapsulated					
	Ctrl		S		Se		Ctrl		S		Se	
**4-O-CQA**												
Raw kale	89.2[1] ± 8.0 a[2]		41.7 ± 3.8 a		68.9 ± 6.2 a		79.2 ± 6.1 a		40.1 ± 3.6 a		59.9 ± 5.4 a	
Mouth	68.6 ± 6.2 b	23%[3]	39.2 ± 3.5 a	6%	53.7 ± 4.8 ab	22%	63.3 ± 5.7 b	20%	34.8 ± 3.1 a	13%	50.5 ± 4.5 b	16%
Stomach	46.3 ± 4.2 c	48%	24.6 ± 2.2 b	41%	39.9 ± 3.6 b	42%	44.4 ± 4.0 c	44%	27.0 ± 2.4 b	32%	41.9 ± 3.8 c	30%
Intestine	11.3 ± 1.0 d	87%	16.3 ± 1.5 c	61%	19.4 ± 1.7 c	72%	29.4 ± 2.6 d	63%	19.0 ± 1.7 c	53%	34.9 ± 3.1 d	42%
**3-O-H-K**												
Raw kale	58.2 ± 5.2 a		41.2 ± 3.7 a		36.6 ± 3.3 a		45.2 ± 4.1 a		37.2 ± 3.3 a		33.6 ± 3.0 a	
Mouth	43.6 ± 3.9 b	25%	33.5 ± 3.0 ab	19%	33.2 ± 3.0 a	9%	37.1 ± 3.3 b	18%	31.6 ± 2.8 b	15%	27.4 ± 2.5 ab	18%
Stomach	24.5 ± 2.2 c	58%	21.8 ± 2.0 b	47%	24.6 ± 2.2 b	33%	23.3 ± 2.1 c	48%	24.1 ± 2.2 c	35%	22.5 ± 2.0 b	33%
Intestine	6.2 ± 0.6 d	89%	15.1 ± 1.4 c	63%	17.0 ± 1.2 c	54%	15.5 ± 1.4 d	66%	19.7 ± 1.8 d	47%	21.5 ± 1.9 b	36%
**Sinapic acid**												
Raw kale	78.3 ± 7.0 a		37.0 ± 3.3 a		50.3 ± 4.5 a		69.3 ± 6.2 a		24.0 ± 2.2 a		43.3 ± 3.9 a	
Mouth	63.0 ± 5.7 ab	19%	30.4 ± 2.7 b	18%	43.2 ± 3.9 a	14%	54.4 ± 4.9 b	21%	19.4 ± 1.7 b	19%	35.8 ± 3.2 a	17%
Stomach	39.6 ± 3.6 b	49%	20.2 ± 1.8 c	45%	26.6 ± 2.4 b	47%	33.8 ± 3.0 c	51%	17.8 ± 1.6 b	26%	29.3 ± 2.6 b	32%
Intestine	8.4 ± 0.8 c	89%	14.4 ± 1.3 d	61%	17.1 ± 1.5 c	66%	27.3 ± 2.5 d	61%	8.8 ± 0.8 c	63%	25.8 ± 2.3 c	40%
**Ferulic acid**												
Raw kale	32.4 ± 2.9 a		29.4 ± 2.6 a		24.6 ± 2.2 a		28.4 ± 2.6 a		27.1 ± 2.4 a		21.6 ± 1.9 a	
Mouth	25.2 ± 2.3 ab	22%	26.4 ± 2.4 a	10%	21.0 ± 1.9 a	15%	24.7 ± 2.2 a	13%	24.1 ± 2.2 a	11%	17.4 ± 1.6 b	20%
Stomach	18.8 ± 1.7 b	42%	18.2 ± 1.6 b	38%	6.5 ± 0.6 b	74%	14.9 ± 1.3 b	48%	20.0 ± 1.8 b	26%	14.1 ± 1.3 c	35%
Intestine	3.8 ± 0.3 c	88%	13.5 ± 1.2 c	54%	8.8 ± 0.8 b	64%	9.6 ± 0.9 c	66%	11.2 ± 1.0 c	59%	10.9 ± 1.0 d	50%

Table S3. (Continuation) Bioaccessibility of individual phenolic compounds in encapsulated and non-encapsulated 7-day-old Red Russian kale sprouts treated with selenium and sulfur.

	Non-Encapsulated												Encapsulated											
	Ctrl				S				Se				Ctrl				S				Se			
**1-S-2-FG**																								
Raw kale	27.1[1]	± 2.4	a[2]		38.0	± 3.4	a		43.4	± 3.9	a		27.1	± 2.4	a		36.7	± 3.3	a		42.4	± 3.8	a	
Mouth	20.1	± 1.8	b	26%[3]	31.6	± 2.8	b	17%	37.6	± 3.4	b	13%	20.7	± 1.9	b	24%	32.2	± 2.9	b	12%	33.0	± 3.0	b	22%
Stomach	6.0	± 0.5	c	78%	10.8	± 1.0	c	72%	17.8	± 1.6	c	59%	11.2	± 1.0	c	59%	26.7	± 2.4	c	27%	28.6	± 2.6	c	32%
Intestine	2.6	± 0.2	cd	90%	6.6	± 0.6	d	83%	15.1	± 1.4	d	65%	7.1	± 0.6	d	74%	15.5	± 1.4	d	58%	23.3	± 2.1	d	45%
**1,2-diS-2-FG**																								
Raw kale	12.0	± 1.1	a		7.3	± 0.7	a		11.6	± 1.0	a		9.0	± 0.8	a		6.0	± 0.5	a		8.6	± 0.8	a	
Mouth	9.5	± 0.9	b	21%	5.0	± 0.4	ab	32%	10.2	± 0.9	a	12%	8.2	± 0.7	a	9%	5.5	± 0.5	a	8%	7.8	± 0.7	a	10%
Stomach	4.8	± 0.4	c	60%	3.4	± 0.3	b	54%	6.1	± 0.5	b	48%	4.0	± 0.4	b	55%	5.2	± 0.5	a	13%	5.0	± 0.5	b	42%
Intestine	0.8	± 0.1	d	94%	1.4	± 0.1	c	80%	2.5	± 0.2	c	79%	2.7	± 0.2	c	70%	2.5	± 0.2	b	59%	3.7	± 0.3	c	57%
**K-3-O-s-so7-O-g**																								
Raw kale	34.1	± 3.1	a		43.8	± 3.9	a		27.0	± 2.4	a		37.1	± 3.3	a		41.1	± 3.7	a		24.0	± 2.2	a	
Mouth	25.7	± 2.3	b	25%	38.5	± 3.5	b	12%	21.6	± 1.9	b	20%	28.7	± 2.6	b	23%	34.5	± 3.1	b	16%	20.9	± 1.9	a	13%
Stomach	7.8	± 0.7	c	77%	18.2	± 1.6	c	58%	9.3	± 0.8	c	66%	24.8	± 2.2	b	33%	29.8	± 2.7	c	28%	15.8	± 1.4	b	34%
Intestine	3.4	± 0.3	d	90%	8.0	± 0.7	d	82%	4.0	± 0.4	d	85%	5.2	± 0.5	c	86%	19.5	± 1.8	d	53%	14.0	± 1.3	b	42%
**Quercetin**																								
Raw kale	24.2	± 2.2	a		39.0	± 3.5	a		24.3	± 2.2	a		19.2	± 1.7	a		38.0	± 3.4	a		23.3	± 2.1	a	
Mouth	17.9	± 1.6	b	26%	29.2	± 2.6	b	25%	19.6	± 1.8	ab	19%	14.3	± 1.3	b	25%	32.9	± 3.0	b	13%	19.7	± 1.8	b	15%
Stomach	5.4	± 0.5	c	77%	17.6	± 1.6	c	55%	8.8	± 0.8	c	64%	8.7	± 0.8	c	54%	25.6	± 2.3	c	33%	14.3	± 1.3	c	39%
Intestine	2.3	± 0.2	d	90%	6.1	± 0.6	d	84%	3.8	± 0.3	d	84%	4.8	± 0.4	d	75%	21.1	± 1.9	d	44%	11.3	± 1.0	d	52%

[1] Concentrations are reported mg per 100 g of kale (DW). [2] Different letters in the same column indicate statistical differences in the concentration of each compound between treatments using the least significant difference (LSD) test ($p < 0.05$). [3] Values in parentheses represent the percentage of degradation with respect to the control. Abbreviations: non-encapsulated (NE), encapsulated (E), selenium (S), sulfur (S), 4-O-caffeoylquinic acid (4-O-CQA), 3-O-hexoside kaempferol (3-O-H-K), 1-sinapoyl-2′-ferulolylgentiobiose (1-S-2-FG), 1,2-disinapoyl-2-ferulolylgentiobiose (1,2-diS-2-FG) and kaempferol 3-*O*-sophoroside-7O-glucoside (K-3-O-s-so7).

Table S4. Bioaccessibility of individual glucosinolates in encapsulated and non-encapsulated 7-day-old Red Russian kale sprouts treated with selenium and sulfur.

	Non-Encapsulated									Encapsulated								
	**Ctrl**			**S**			**Se**			**Ctrl**			**S**			**Se**		
**GIB**																		
Raw kale	0.1[1] ± 0.0	a[2]		4.0 ± 0.4	a		1.2 ± 0.1	a		ND	a		2.4 ± 0.2	a		0.5 ± 0.0	a	
Mouth	ND	b	100%[3]	3.1 ± 0.3	b	23%	1.0 ± 0.1	b	16%	ND	a		2.1 ± 0.2	a	15%	0.4 ± 0.0	a	12%
Stomach	ND	b	100%	2.1 ± 0.2	c	46%	0.7 ± 0.1	c	41%	ND	a		1.4 ± 0.1	b	41%	0.3 ± 0.0	b	39%
Intestine	ND	b	100%	ND	d	100%	ND	d	100%	ND	a		ND	c	100%	ND	c	100%
**PRO**																		
Raw kale	1.9 ± 0.2	a		3.6 ± 0.3	a		2.0 ± 0.2	a		1.6 ± 0.1	a		2.2 ± 0.2	a		1.4 ± 0.1	a	
Mouth	1.5 ± 0.1	a	21%	3.1 ± 0.3	ab	12%	1.8 ± 0.2	a	10%	1.4 ± 0.1	a	11%	1.9 ± 0.2	a	10%	1.3 ± 0.1	a	9%
Stomach	1.0 ± 0.1	b	45%	2.2 ± 0.2	c	39%	1.3 ± 0.1	b	37%	1.0 ± 0.1	b	38%	1.5 ± 0.1	b	33%	0.9 ± 0.1	b	36%
Intestine	ND	c	100%	ND	d	100%	ND	c	100%	0.3 ± 0.0	c	81%	0.6 ± 0.1	c	71%	0.5 ± 0.0	c	65%
**GRA**																		
Raw kale	24.7 ± 2.2	a		157.1 ± 14.1	a		111.5 ± 10	a		23.5 ± 2.1	a		149.6 ± 13.5	a		103.5 ± 9.3	a	
Mouth	18.8 ± 1.7	b	24%	138.5 ± 12.5	b	12%	93.8 ± 8.4	a	16%	18.8 ± 1.7	a	20%	132.2 ± 11.9	a	12%	88.9 ± 8.0	a	14%
Stomach	13.1 ± 1.2	c	47%	97.0 ± 8.7	c	38%	84.4 ± 7.6	b	24%	15.2 ± 1.4	b	35%	99.3 ± 8.9	b	34%	80.1 ± 7.2	b	23%
Intestine	12.3 ± 1.1	c	50%	82.9 ± 7.5	d	47%	75.1 ± 6.8	b	33%	6.5 ± 0.6	c	72%	85.0 ± 7.7	c	43%	73.3 ± 6.6	b	29%
**GNP**																		
Raw kale	8.9 ± 0.8	a		9.5 ± 0.9	a		11.6 ± 1.0	a		6.9 ± 0.6	a		8.6 ± 0.8	a		10.6 ± 1.0	a	
Mouth	6.3 ± 0.6	b	29%	8.6 ± 0.8	b	9%	9.2 ± 0.8	a	21%	5.1 ± 0.5	a	26%	7.9 ± 0.7	a	8%	9.5 ± 0.9	b	11%
Stomach	4.4 ± 0.4	c	50%	6.0 ± 0.5	c	37%	6.5 ± 0.6	b	44%	4.6 ± 0.4	b	34%	6.4 ± 0.6	b	25%	6.6 ± 0.6	c	37%
Intestine	ND	d	100%	ND	d	100%	ND	c	100%	2.0 ± 0.2	c	72%	2.8 ± 0.2	c	68%	2.9 ± 0.3	d	73%

Table S4. (Continuation) Bioaccessibility of individual glucosinolates in encapsulated and non-encapsulated 7-day-old Red Russian kale sprouts treated with selenium and sulfur.

	Non-Encapsulated						Encapsulated					
	Ctrl		S		Se		Ctrl		S		Se	
**4-HGB**												
Raw kale	0.1[1] ± 0.0 a[2]		0.7 ± 0.1 a		0.4 ± 0.0 a		ND a		0.4 ± 0.0 a		1.2 ± 0.1 a	
Mouth	0.1 ± 0.0 a	20%[3]	0.4 ± 0.0 b	49%	ND b	100%	ND a		0.3 ± 0.0 b	67%	1.1 ± 0.1 b	10%
Stomach	0.0 ± 0.0 b	56%	0.3 ± 0.0 b	65%	ND b	100%	ND a		ND c	100%	0.8 ± 0.1 c	37%
Intestine	ND c	100%	ND c	100%	ND b	100%	ND a		ND c	100%	ND d	100%
**GER**												
Raw kale	0.1 ± 0.0 a		0.2 ± 0.0 a		0.4 ± 0.0 a		ND a		0.5 ± 0.0 a		0.4 ± 0.0 a	
Mouth	0.1 ± 0.0 a	27%	0.2 ± 0.0 a	14%	0.3 ± 0.0 b	21%	ND a		0.5 ± 0.0 a	9%	0.2 ± 0.0 a	34%
Stomach	0.1 ± 0.0 a	76%	0.1 ± 0.0 b	45%	0.1 ± 0.0 c	52%	ND a		0.3 ± 0.0 b	37%	ND b	100%
Intestine	ND a	100%	ND c	100%	ND d	100%	ND a		ND c	100%	ND c	100%
**GBS**												
Raw kale	5.0 ± 0.5 a		20.8 ± 1.9 a		10.8 ± 1.0 a		2.7 ± 0.2 a		16.1 ± 1.5 a		9.5 ± 0.9 a	
Mouth	3.7 ± 0.3 b	26%	16.5 ± 1.5 b	21%	8.2 ± 0.7 b	24%	2.0 ± 0.2 a	24%	14.0 ± 1.3 a	13%	8.6 ± 0.8 a	9%
Stomach	3.4 ± 0.3 b	33%	13.2 ± 1.2 c	37%	7.8 ± 0.7 b	28%	1.8 ± 0.2 b	31%	11.2 ± 1.0 b	30%	6.0 ± 0.5 b	36%
Intestine	2.0 ± 0.2 c	60%	12.0 ± 1.1 c	42%	4.7 ± 0.4 c	56%	1.6 ± 0.1 b	38%	9.3 ± 0.8 c	42%	5.9 ± 0.5 b	38%
**4-MGBS**												
Raw kale	16.4 ± 1.5 a		33.6 ± 3.0 a		59.5 ± 5.4 a		14.5 ± 1.3 a		28.5 ± 2.6 a		49.2 ± 4.4 a	
Mouth	12.5 ± 1.1 b	24%	27.6 ± 2.5 b	18%	48.5 ± 4.4 b	18%	11.9 ± 1.1 a	18%	23.3 ± 2.1 a	19%	41.9 ± 3.8 b	15%
Stomach	8.8 ± 0.8 c	47%	19.3 ± 1.7 c	43%	34.0 ± 3.1 c	43%	9.3 ± 0.8 b	36%	18.4 ± 1.7 b	35%	32.4 ± 2.9 c	34%
Intestine	4.1 ± 0.4 d	75%	14.4 ± 1.3 d	57%	9.3 ± 0.8 d	84%	4.0 ± 0.4 c	72%	14.4 ± 1.3 c	49%	13.9 ± 1.3 d	72%

[1] Concentrations are reported mM per 100 g of kale (DW). [2] Values in parentheses represent the percentage of degradation with respect to the control. [3] Different letters in the same column indicate statistical differences in the concentration of each compound between treatments using the least significant difference (LSD) test ($p < 0.05$). Abbreviations: ND (No detected), non-encapsulated (NE), encapsulated (E), selenium (S), sulfur (S), glucoiberin (GIB), progoitrin (PRO), glucoraphanin (GRA), gluconapin (GNP), 4-hydroxy-glucobrassicin (4-HGB), glucoeurocin (GER), glucobrassicin (GBS) and 4-methoxy-glucobrassicin (4-MGBS).

1. Cellular antioxidant activity (CAA)

To evaluate the cellular antioxidant activity of intestinal-digested fractions of encapsulated and non-encapsulated kale sprouts, the method described by Ortega-Hernández et. al., *[1]* was used. Human colorectal adenocarcinoma cells (Caco-2) were obtained from the American Type Culture Collection (ATCC® TIB-71™, VA, USA). Cells were cultivated in a DMEM solution containing 5% fetal bovine serum and 1% Pen-Strep antibiotic at 37 °C and 5% CO_2. Cells were seeded at a density of 5×10^4 cells/well on a 96-well plate and allowed to adhere for 16 h. Afterward, cells were treated with 100 µL of intestinal-digested fractions (200, 240, 300, and 400 µg/mL) of encapsulated and non-encapsulated kale treated with Se and S containing DCFH-DA (60 µM). After removing the treatment solutions, the cells were rinsed twice with PBS. Lastly, 100 µL of a 500 µM AAPH solution was added to each well, excluding the wells serving as blank and negative controls. Fluorescence was measured at 538 nm (emission) and 485 nm (excitation) every 2 min for 90 min at 37 ºC using a microplate reader. Equation 3 was utilized to calculate CAA values:

$$CAA\ Unit = 1 - \left(\frac{\int SA}{\int CA}\right) \quad (1)$$

where $\int$ SA represents the integrated area under the curve of sample fluorescence versus time, and $\int$ CA represents the integrated area from the control curve.

2. Evaluation of the anti-inflammatory potential by nitric oxide determination

The production of nitric oxide was evaluated using the method described by Ortega-Hernández et al., [1].

2.1. Cell culture

Murine macrophages Raw 264.7 cells and human colorectal adenocarcinoma cells (Caco-2) were obtained from the American Collection Type Culture (ATCC® TIB-71™, VA, USA). The cells were inoculated in a DMEM solution supplemented with 5% fetal bovine serum and 1% Pen-Strep antibiotic at 37 °C and 5% CO_2. Cells were seeded in a 96-well plate (5×10^4 cells/well) and allowed to adhere for 16 h to evaluate the effects of intestinal-digested fractions of kale on cellular antioxidant activity. After that, intestinal-digested fractions of encapsulated and non-encapsulated kale were added to Raw 264.7 (10, 25, and 50 µg/mL) and Caco-2 cells (200, 240, and 400 µg/mL), respectively. After 4 h of incubation, half of the wells were stimulated with LPS at 1 µg/mL, while the remaining wells served as controls for each sample.

2.2. Measurement of nitric oxide

The nitrite concentration in the cell culture supernatant was used as a measure of nitric oxide (NOx) production. The amount of nitrite in the medium (100 µL) was measured with the Griess Reagent System (Promega, Madison, WI) according to the manufacturer's directions. The absorbance readings were obtained at 550 nm on a Synergy HT plate reader (Bio-Tek Instruments, Inc., VT, USA) after 10 min of incubation. A standard curve of sodium nitrite (1.5-50 µM) was prepared to quantify nitrate concentration.

2.3. Measurement of Raw 264.7 cell viability

Cell viability was tested using the CellTiter 96 AQueous One Solution Cell Proliferation Assay (Promega, Madison, WI). Absorbance values were read with a 96-well microplate reader (Synergy HT, Bio-Tek, Winooski, VM) at 490 nm. The percentage (%) of cell viability was calculated by dividing the absorbance of treated cells by the absorbance of the control (untreated) cells.

References

1. Ortega-Hernández, E.; Acevedo-Pacheco, L.; Jacobo-Velázquez, D.A.; Antunes-Ricardo, M. Bioaccessibility and Potential Biological Activities of Lutein, Glucosinolates, and Phenolic

Compounds Accumulated in Kale Sprouts Treated with Selenium, Sulfur, and Methyl Jasmonate. *ACS Food Sci. Technol.* **2023**, *3*, 404–413, doi:10.1021/acsfoodscitech.2c00285.
